# Dynamic Response of EPS Foam in Packaging: Experimental Tests and Constitutive Modeling

**DOI:** 10.3390/polym17121606

**Published:** 2025-06-09

**Authors:** Pei Li, Heng Zhang, Leilei Chen

**Affiliations:** 1 Centre for Industrial Mechanics, Institute of Mechanical and Electrical Engineering, University of Southern Denmark, 6400 Sønderborg, Denmark; 2 International Machinery Center, School of Mechanical Engineering, Xi’an Jiaotong University, Xi’an 710049, China; zhangheng@stu.xjtu.edu.cn; 3 Henan International Joint Laboratory of Structural Mechanics and Computational Simulation, School of Architecture and Civil Engineering, Huanghuai University, Zhumadian 463000, China; chenllei@mail.ustc.edu.cn

**Keywords:** EPS foam, TV package, drop test, dynamic response, rate sensitive constitutive model

## Abstract

Expanded polystyrene (EPS) foam is widely used in energy-absorbing structures for packaging applications; however, its mechanical behavior under dynamic loading conditions remains insufficiently characterized. To address this, the dynamic responses of EPS foam used in television packaging were first examined experimentally through drop tests. Building on these findings, a rate-sensitive constitutive model was developed to incorporate tensile damage mechanisms and tension–compression asymmetry, enabling unified modeling of both tensile and compressive deformation in complex structural applications. The proposed model was calibrated using standardized tension, compression, and shear tests, and subsequently employed to simulate three-point bending and dynamic compression scenarios involving EPS foam components. The simulation results demonstrated favorable agreement with experimental observations, confirming the accuracy and robustness of the proposed constitutive model in predicting the dynamic mechanical behavior of EPS foam.

## 1. Introduction

Expanded polystyrene (EPS) foam is widely utilized across various industries due to its distinctive characteristics, including low density, excellent energy absorption, and low thermal conductivity [1,2,3,4,5]. Its primary application lies in packaging, where it serves as a liner or filling material, benefiting from its superior energy absorption under impact loading [6]. However, accurately characterizing the mechanical behavior of EPS foam remains a significant challenge due to its complex and randomly distributed cellular structure.

To better understand the mechanical response of EPS foam, both static and dynamic experiments have been conducted over the past decades. For example, Ouellet [7] examined the dynamic compressive behavior at a strain rate of approximately 2500 s^−1^, demonstrating that both the plateau stress and densification strain are strain rate-sensitive and dependent on foam density. In tension, EPS foam exhibits creep behavior influenced by both applied stress and density, which can be effectively described using viscoelastic models [8]. Zou [9] studied the time-dependent creep behaviors of EPS geofoam, and the data obtained were employed to calibrate the viscoelastic models, giving an accurate prediction of mechanical performance. Park [10] analyzed the time-dependent creep behavior of EPS foam, and the computational accuracy of FEA model will be improved when the creep properties are included. Chen [6] subsequently conducted a comprehensive study on the compressive and tensile behaviors of EPS foams with densities of 13.5 kg/m^3^ and 28 kg/m^3^, revealing pronounced rate sensitivity in their mechanical properties through experimental testing.

In packaging applications, fatigue resistance is also critical. Ozturk [11,12] investigated the response of EPS foam under repeated compressive loading and unloading, showing that both energy absorption and absorption efficiency decrease with the number of loading cycles. Nevertheless, EPS foam is often subjected to more complex multiaxial loading conditions in real-world scenarios, necessitating further investigation into its behavior under varying stress states and loading rates. For instance, Farajzadeh [13] studied EPS foam under biaxial loading and observed that shear stiffness increases with axial strain, resulting in higher shear stresses. Similar findings were reported by Arnesen [14], who analyzed foam response under V-shaped impact conditions and noted that higher-density EPS foam exhibited reduced elastic rebound and increased residual deformation under shear.

Ling [15,16] further explored the coupled compression–shear response of EPS foam under both static and dynamic loading using a novel experimental system capable of decoupling compressive and shear effects. Their results indicated that the yield stress under combined loading could be up to 40% lower than that under uniaxial compression and that it decreases further with increasing impact angle, depending on both foam density and strain rate. Krundaeva [17] investigated the dynamic compression of spherical EPS foam to simulate helmet applications. A related study by Kumar [18] modeled the performance of EPS foam in actual bicycle helmets subjected to lateral impacts on flat and hemispherical anvils. More recently, Yan [19] examined the impact response of EPS foam in rockfall protection galleries, focusing on fracture and energy dissipation mechanisms. Although these studies provide a comprehensive understanding of the mechanical behavior of EPS foam under uniaxial and biaxial loading, there is a notable lack of research specifically addressing its dynamic response in the context of packaging engineering, the most prevalent application scenario.

To address these challenges, several constitutive models have been proposed in the literature. For example, Tang [20] utilized a linear fitting constitutive model to simulate three-point bending tests. In this approach, the tensile response was assumed to follow linear elasticity, while the compressive response was modeled using a bilinear phenomenological representation. However, this simplified formulation falls short in capturing the inherent nonlinearities of EPS foam. To better represent the rate-dependent and nonlinear behavior, Zouzias [21] employed the Avalle constitutive model [22]—originally designed for compressive behavior—to predict the tensile response of EPS foam. While it offered some improvements, its predictive accuracy remained limited due to the model’s original focus on compressive regimes. Rodríguez-Sánchez [23] explored the hyperfoam model under various strain rates and demonstrated its ability to accurately capture the energy absorption characteristics of EPS foam in compression.

With the advent of machine learning, data-driven models have emerged as powerful tools for predicting foam behavior. Rodríguez-Sánchez [24,25] investigated how loading rate and foam density influence compressive response and hysteresis, while Pech-Mendoza [26] examined the role of bead size distribution on the mechanical response, further enhancing predictive modeling capabilities. However, as noted by Arnesen [14], several built-in constitutive models in commercial simulation software (e.g., LS-DYNA) fail to reproduce the complex response of EPS foam under combined compression–shear loading conditions. Many traditional models either oversimplify the deformation mechanisms or rely on parameters that are difficult to calibrate experimentally.

To overcome these limitations, anisotropic constitutive models have shown increasing promise in capturing the directional dependencies inherent in cellular materials [27,28,29]. For example, refs. [30,31] studied the anisotropic compressive response of polyurethane foam and developed a general rate-sensitive model to describe its mechanical performance. Building on this foundation, the present study aims to enhance an anisotropic constitutive framework to account for the tension–compression asymmetry and tensile damage observed in EPS foam, particularly under dynamic loading scenarios. The proposed model is validated through drop tests simulating packaging conditions, providing a comprehensive assessment of its practical applicability.

## 2. Dynamic Compression Experiments of EPS Foam

### 2.1. Dynamic Compression of Non-Standard Specimens

**Figure 1:**
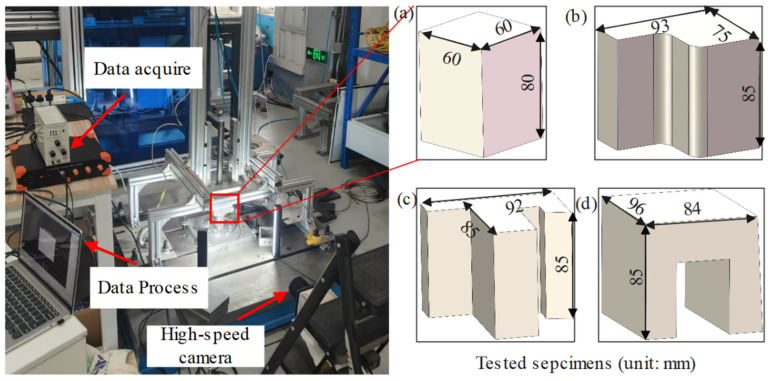
Experimental arrangement of dynamic compression tests and different tested specimens: (**a**) standard specimen; (**b**) non-standard specimen 1; (**c**) non-standard specimen 2; and (**d**) non-standard specimen 3.

In this study, a commercially available EPS18 foam with a nominal density of 18.1 kg/m^3^ was used. The foam was manually machined into four representative specimen types incorporating different geometric features, as illustrated in Figure 1a–d. These include a standard specimen and three non-standard configurations, designed to reflect typical structural features encountered in engineering applications. Dynamic compression tests were conducted using a drop hammer apparatus, also shown in Figure 1. The hammer was released from a height of approximately 1.1 m, corresponding to an impact velocity of about 4.5 m/s. During each test, the specimen was placed on a rigid base equipped with a load cell to record the transmitted compressive force. Additionally, a high-speed camera operating at 2600 frames per second was used to capture the deformation process for subsequent analysis.

**Figure 2:**
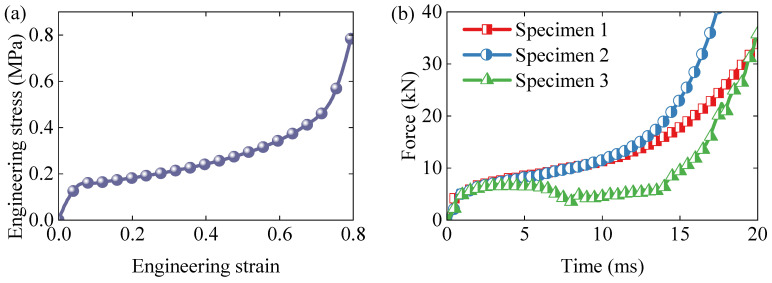
Results of dynamic compression tests: (**a**) stress–strain curve of standard specimen; (**b**) force–time responses of non-standard specimens.

The engineering stress–strain curve of the standard specimen, shown in Figure 2a, reveals the typical three-stage response of EPS foam: an initial linear elastic region, followed by a plateau region associated with progressive cell collapse, and concluding with a densification stage. The measured overall elastic stiffness, yield stress, and densification strain were approximately 3.47 MPa, 0.153 MPa, and 0.70 MPa, respectively.

Figure 2b presents the force–time responses of the non-standard specimens. Specimen 1 and specimen 2 displayed similar profiles; however, specimen 2 entered the densification stage earlier, likely due to structural differences. In contrast, specimen 3 exhibited a distinct response: a brief elastic stage followed by a shortened plateau and a subsequent force drop. This behavior is attributed to damage at the “L”-shaped corner of the specimen. The deformation mechanisms and failure modes of these geometries are discussed in detail in Section 4.3.

### 2.2. Dynamic Compression of TV Package

**Figure 3:**
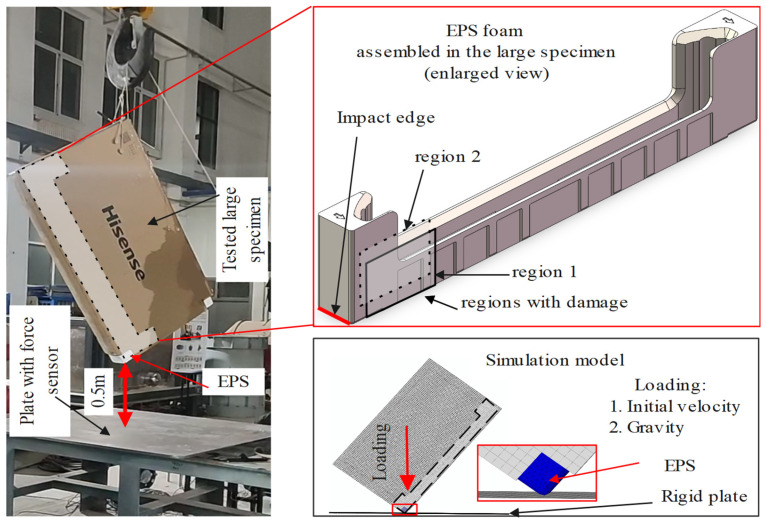
Experimental setup for TV package drop test and corresponding simulation model.

To further investigate the mechanical performance of EPS foam in realistic packaging applications, dynamic impact experiments were conducted on a full-scale television (TV) packaging system. The assembled package, with dimensions of 1400 mm × 830 mm × 125 mm, consisted of two EPS foam components, a corrugated cardboard shell, and a dummy TV model. The entire assembly was lifted to a height of approximately 0.5 m and then released in free fall to simulate impact loading. To specifically assess the performance of EPS foam under localized impact, the bottom-right corner of the EPS component was deliberately exposed, allowing for direct contact with a rigid base plate, as illustrated in Figure 3. The base was instrumented with four force sensors embedded within the plate to record the transmitted impact force. A total of six repeated drop tests were performed to minimize experimental variability. For each trial, the EPS foam component was replaced to avoid material fatigue, while the rest of the assembly remained unchanged to maintain consistency.

**Figure 4:**
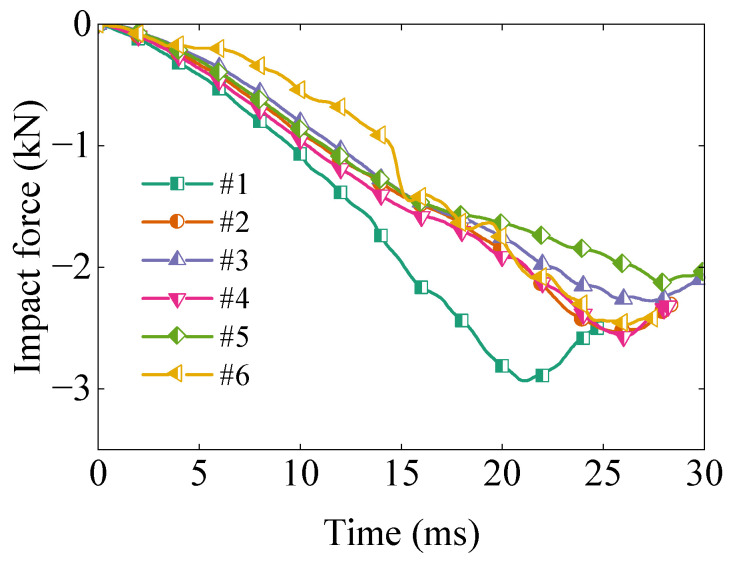
Measured impact force–time curves from six repeated TV package drop tests.

Figure 4 presents the impact force–time curves obtained from the six tests. The first drop tests yielded the highest peak force, maybe due to variation in impact direction. However, the peak values stabilized in subsequent trials. Specifically, tests #2, #4, and #6 exhibited nearly identical peak forces. These three datasets were used to compute the average impact force, serving as the representative result for subsequent analysis.

## 3. Rate-Sensitive Constitutive Model

To predict the deformation behavior of EPS foam under dynamic loading, the rate-sensitive anisotropic constitutive model previously proposed by the authors [1] was briefly reviewed in this section and then modified for application to EPS foam. The model accounts for anisotropic elasticity, strain rate dependence, and anisotropic post-yield hardening, characteristics essential for capturing the complex mechanical response of foam materials under impact conditions.

### 3.1. Brief Review of Rate-Sensitive Anisotropic Model (Li-Guo-Shim Model)

#### 3.1.1. Anisotropic Elastic Constitutive Model

The total strain rate $\dot{\varepsilon }$ can be decomposed into elastic component ${\dot{\varepsilon }}^{e}$ and plastic one ${\dot{\varepsilon }}^{p}$ as shown as follows:

(1) $$\dot{\varepsilon }={\dot{\varepsilon }}^{e}+{\dot{\varepsilon }}^{p}$$

The Cauchy stress rate $\dot{\sigma }$ is then related to the strain rate via the elastic stiffness tensor C:

(2) $$\dot{\sigma }=C\left(\dot{\varepsilon }-{\dot{\varepsilon }}^{p}\right)$$

For isotropic materials, the stiffness tensor C is characterized by only two independent parameters (Young’s modulus and Poisson’s ratio). However, for anisotropic materials, additional parameters are needed.

#### 3.1.2. Anisotropic Yield Function

To capture anisotropic yielding, Tagarielli [32] proposed a generalized yield function for transversely isotropic materials:

(3) $$\bar{f}=\bar{\sigma }-Y=\sqrt{\sigma^{T}Q\sigma }-Y=0$$

The matrix Q defines the transversely isotropic yield response and is expressed as follows:

(4) $$Q=\left[\begin{array}{cccccc} B^{2} & {-C}^{2}/2 & {-D}^{2}/2 & 0 & 0 & 0\\ {-C}^{2}/2 & B^{2} & {-D}^{2}/2 & 0 & 0 & 0\\ {-D}^{2}/2 & {-D}^{2}/2 & 1 & 0 & 0 & 0\\ 0 & 0 & 0 & E^{2} & 0 & 0\\ 0 & 0 & 0 & 0 & F^{2} & 0\\ 0 & 0 & 0 & 0 & 0 & F^{2} \end{array}\right]$$

where direction 3 is selected as a reference direction in a Cartesian coordinate system (1, 2, 3). B, C, D, E, and F are five constants determined by the transversely isotropic yield behavior.

The plastic strain rate is then defined using the associated flow rule:

(5) $${\dot{\varepsilon }}^{p}={\dot{\bar{\varepsilon }}}^{p}\frac{\partial \bar{f}}{\partial \sigma }$$

where the ${\dot{\bar{\varepsilon }}}^{p}$ is the effective plastic strain rate defined as ${\dot{\bar{\varepsilon }}}^{p}=\sqrt{{\dot{\varepsilon }}^{pT}Q^{-1}{\dot{\varepsilon }}^{p}}$.

However, as noted in reference [33], this model captures only transversely isotropic yield and does not account for anisotropic post-yield hardening or strain-rate effects. To address these limitations, the authors proposed an enhanced yield function [1]:

(6) $$\tilde{f}=\tilde{\sigma }-Y=\sqrt{\sigma^{T}H^{-T}QH^{-1}\sigma }-Y=\sqrt{{\hat{\sigma }}^{T}Q\hat{\sigma }}-Y=0$$

where H represents the anisotropic hardening matrix, while Q is the fully anisotropic yield tensor, as follows:

(7) $$Q=\left[\begin{array}{cccccc} Q_{1111} & Q_{1122} & Q_{1133} & Q_{1112} & Q_{1123} & Q_{1131}\\ Q_{2211} & Q_{2222} & Q_{2233} & Q_{2212} & Q_{2223} & Q_{2231}\\ Q_{3311} & Q_{3322} & Q_{3333} & Q_{3312} & Q_{3323} & Q_{3331}\\ Q_{1211} & Q_{1222} & Q_{1233} & Q_{1212} & Q_{1223} & Q_{1231}\\ Q_{2311} & Q_{2322} & Q_{2333} & Q_{2312} & Q_{2323} & Q_{2331}\\ Q_{3111} & Q_{3122} & Q_{3133} & Q_{3112} & Q_{3123} & Q_{3131} \end{array}\right]$$

This tensor also exhibits symmetry; i.e., $Q_{ijkl}=Q_{klij}=Q_{jikl}$. The hardening matrix H is simplified as a diagonal matrix for a simplify implementation.

(8) $$H=\left[\begin{array}{cccccc} h_{11} & & & & & \\ & h_{22} & & & & \\ & & h_{33} & & & \\ & & & h_{12} & & \\ & & & & h_{23} & \\ & & & & & h_{13} \end{array}\right]$$

Each diagonal component of H, accounting for rate-dependent hardening, is expressed as follows:

(9) $$h_{ij}\left({\bar{\varepsilon }}^{p},\dot{\varepsilon }\right)=\frac{Y_{ij}\left({\bar{\varepsilon }}^{p},\dot{\varepsilon }\right)}{Y_{ij}^{0}\left(0,{\dot{\varepsilon }}_{0}\right)}$$

where ${\dot{\varepsilon }}_{0}$ denotes a reference strain rate and ${\bar{\varepsilon }}^{p}$ is the conventional effective plastic strain (see Equation (5)).

For ease of experimental characterization and numerical implementation, the strain-rate effect and post-yield hardening are assumed to be independent, and Equation (9) is further decomposed as follows:

(10) $$h_{ij}\left({\bar{\varepsilon }}^{p},\dot{\varepsilon }\right)=\frac{Y_{ij}\left({\bar{\varepsilon }}^{p},{\dot{\varepsilon }}_{0}\right)}{Y_{ij}^{0}\left(0,{\dot{\varepsilon }}_{0}\right)}\frac{Y_{ij}\left(0,\dot{\varepsilon }\right)}{Y_{ij}^{0}\left(0,{\dot{\varepsilon }}_{0}\right)}$$

The first term captures anisotropic post-yield hardening at the reference strain rate, while the second describes strain rate sensitivity at the yield point.

#### 3.1.3. Plastic Flow Rule

When the associated flow rule is adopted, the plastic strain rate ${\dot{\varepsilon }}^{p}$ is assumed to be normal to the yield surface and defined by the following:

(11) $${\dot{\varepsilon }}^{p}=\dot{\lambda }\frac{\partial f}{\partial \sigma }/\left|\frac{\partial f}{\partial \sigma }\right|$$

For plastic work conjugacy, the plastic work rate is computed from the modified effective plastic strain rate ${\dot{\tilde{\varepsilon }}}^{p}$ and the modified effective stress $\tilde{\sigma }$, equal to the work calculated by the Cauchy stress σ and plastic strain rate ${\dot{\varepsilon }}^{p}$.

(12) $$\tilde{\sigma }{\dot{\tilde{\varepsilon }}}^{p}=\sigma {\dot{\varepsilon }}^{p}$$

The effective plastic strain rate ${\dot{\tilde{\varepsilon }}}^{p}$ is defined as follows (see ref. [1] for derivation details):

(13) $${\dot{\tilde{\varepsilon }}}^{p}=\sqrt{{\dot{\varepsilon }}^{pT}H^{T}Q^{-1}H{\dot{\varepsilon }}^{p}}$$

By substituting Equations (12) and (13) into Equation (11), the plastic multiplier $\dot{\lambda }$ is obtained as follows:

(14) $$\dot{\lambda }={\dot{\tilde{\varepsilon }}}^{p}\left|\frac{\partial f}{\partial \sigma }\right|$$

Therefore, the plastic strain rate at the yield point can be derived as follows:

(15) $${\dot{\varepsilon }}^{p}={\dot{\tilde{\varepsilon }}}^{p}\frac{\partial f}{\partial \sigma }={\dot{\tilde{\varepsilon }}}^{p}\frac{Q\sigma }{\tilde{\sigma }}$$

#### 3.1.4. Damage Model

The mechanical response of EPS foam under tension differs significantly from that under compression. In particular, brittle fracture is a prominent feature of the tensile response [7]. To accurately capture this behavior, a damage criterion was incorporated into the constitutive model.

Damage is initiated when the maximum principal strain $\varepsilon_{mp}$ reaches the tensile failure strain $\varepsilon_{f}$:

(16) $$\varepsilon_{mp}-\varepsilon_{f}=0$$

To mitigate mesh dependency, a displacement-based damage evolution model was adopted. A simple linear evolution law was used:

(17) $$\dot{d}=\left\{\begin{array}{cc} 0 & \;,if\;\varepsilon_{mp}<\varepsilon_{f}\;or\;d=1\\ \frac{\dot{\bar{u}}}{{\bar{u}}_{f}}=\frac{L_{c}{\dot{\varepsilon }}_{mp}}{{\bar{u}}_{f}} & ,if\;\varepsilon_{mp}\geq \varepsilon_{f}\;and\;d\leq 1 \end{array}\right.$$

where $\dot{d}$ denotes the rate of change of the damage parameter d, which ranges from 0 (no damage) to 1 (complete failure). $\dot{\bar{u}}$ is the rate of displacement, and ${\bar{u}}_{f}$ is the critical displacement from damage initiation to complete failure. $L_{c}$ denotes the characteristic length of elements.

The Cauchy stress tensor incorporating damage is expressed as follows:

(18) $$\overset{˘}{\sigma }=\left(1-d\right)\sigma$$

### 3.2. Simplifying for Quasi-Isotropic Model and Its Implementation

To determine the diagonal components of the anisotropic yield tensor Q, six basic modes of loading (i.e., three uniaxial loading and three simple shear loading) are considered at the onset of yield (i.e., $\varepsilon^{p}=0$). Under these conditions, the anisotropic hardening matrix H is taken as the identity matrix $I_{6\times 6}$. For these uniaxial loading cases, only one Cauchy stress component $\sigma_{ij}$ is non-zero and its value equals to the yield stress $Y_{ij}^{0}$, resulting in a simple yield function:

(19) $$\tilde{f}=\sqrt{\sigma_{ij}I_{ijij}Q_{ijij}I_{ijij}\sigma_{ij}}-Y_{33}^{0}=Y_{ij}^{0}\sqrt{Q_{ijij}}-Y_{33}^{0}=0$$

This leads to the following equation for determining the six diagonal constants:

(20) $$Q_{ijij}={\left(\frac{Y_{33}^{0}}{Y_{ij}^{0}}\right)}^{2}$$

To determine the non-diagonal elements in tensor Q, the plastic Poisson’s ratio is introduced as $\upsilon_{ijkl}^{p}=-\frac{{\dot{\varepsilon }}_{kl}^{p}}{{\dot{\varepsilon }}_{ij}^{p}}$. For a simple mode of loading (e.g., uniaxial compression in 1 direction), the plastic Poisson’s ratio becomes the following:

(21) $$\upsilon_{11kl}^{p}=-\frac{{\dot{\varepsilon }}_{kl}^{p}}{{\dot{\varepsilon }}_{11}^{p}}$$

Substituting Equation (15) into Equation (21) yields the following:

(22) $$\upsilon_{11kl}^{p}=-\frac{{\dot{\tilde{\varepsilon }}}^{p}\frac{Q_{kl11}Y_{11}^{0}}{\tilde{\sigma }}}{{\dot{\tilde{\varepsilon }}}^{p}\frac{Q_{1111}Y_{11}^{0}}{\tilde{\sigma }}}=-\frac{Q_{kl11}}{Q_{1111}}$$

indicating that $Q_{kl11}=Q_{11kl}={-Q_{1111}\upsilon }_{11kl}^{p}$. This derivation also applies to the other five basic modes of loading and leads to a general expression for determining the non-diagonal elements in tensor Q.

(23) $$Q_{ijkl}={-Q_{ijij}\upsilon }_{ijkl}^{p}$$

Assuming negligible coupling between normal and shear strains, and between different shear components, the anisotropic yield tensor Q can be further simplified as follows:

(24) $$Q=\left[\begin{array}{cccccc} Q_{1111} & Q_{1122} & Q_{1133} & 0 & 0 & 0\\ Q_{2211} & Q_{2222} & Q_{2233} & 0 & 0 & 0\\ Q_{3311} & Q_{3322} & Q_{3333} & 0 & 0 & 0\\ 0 & 0 & 0 & Q_{1212} & 0 & 0\\ 0 & 0 & 0 & 0 & Q_{2323} & 0\\ 0 & 0 & 0 & 0 & 0 & Q_{3131} \end{array}\right]$$

where the elements in tensor Q are determined by Equations (20) and (23).

The EPS foam studied in this work exhibits quasi-isotropic yield and post-yield responses, i.e., $Y_{12}=Y_{23}=Y_{31}$, and $Y_{11}=Y_{22}=Y_{33}$, which is not exactly an isotropic response according to the definition of the anisotropy of a material’s mechanical behavior. Due to the near zero Poisson’s ratio of EPS foam [19], the yield tensor Q can finally be re-written as follows:

(25) $$Q=\left[\begin{array}{cccccc} 1 & 0 & 0 & & & \\ 0 & 1 & 0 & & & \\ 0 & 0 & 1 & & & \\ & & & {\left(Y_{33}^{0}/Y_{12}^{0}\right)}^{2} & & \\ & & & & {\left(Y_{33}^{0}/Y_{12}^{0}\right)}^{2} & \\ & & & & & {\left(Y_{33}^{0}/Y_{12}^{0}\right)}^{2} \end{array}\right]$$

To determine the incremental equivalent plastic strain, a Newton–Raphson iterative approach is used:

(26) $$\Delta {\tilde{\varepsilon }}_{k+1}^{p}=\Delta {\tilde{\varepsilon }}_{k}^{p}-\frac{f\left({\tilde{\varepsilon }}_{t}^{p}+\Delta {\tilde{\varepsilon }}_{k}^{p}\right)}{\partial f/\partial \left(\Delta {\tilde{\varepsilon }}^{p}\right)}$$

where the expression of $\partial f/\partial \left(\Delta {\tilde{\varepsilon }}^{p}\right)$ was derived in reference [30] and given as follows:

(27) $$\frac{\partial f}{\partial \left(\Delta {\tilde{\varepsilon }}^{p}\right)}=\frac{{\hat{\sigma }}_{t+\Delta t}^{T}Q}{\tilde{\sigma }}\left[-H_{t+\Delta t}^{-1}H_{t+\Delta t}^{-1}\left(\frac{\partial H_{t+\Delta t}}{\partial \left(\Delta {\bar{\varepsilon }}^{p}\right)}+\frac{\partial H_{t+\Delta t}}{\partial \left(\Delta \dot{\varepsilon }\right)}\frac{1}{\Delta t}\right)\frac{\tilde{\sigma }}{\bar{\sigma }}\sigma_{t+\Delta t}-H_{t+\Delta t}^{-1}C\frac{\partial \tilde{f}}{\partial \sigma }\right]$$

Due to quasi-isotropy, the diagonal dimensionless hardening functions (corresponding to uniaxial loadings) in the hardening matrix ***H*** (see Equation (8)) are simplified as follows:

(28) $$h_{11}=h_{22}=h_{33}=\frac{Y_{11}}{Y_{11}^{0}}k_{11}\left(\frac{\dot{\varepsilon }}{{\dot{\varepsilon }}_{0}}\right)$$

where $Y_{11}$ is the flow stress for uniaxial loading in 1 direction, and the rate-sensitivity term $k_{11}\left(\frac{\dot{\varepsilon }}{{\dot{\varepsilon }}_{0}}\right)$ obtained from uniaxial tests under various strain rates. Similarly, shear components in the hardening matrix ***H*** are derived as follows:

(29) $$h_{12}=h_{23}=h_{31}=\frac{Y_{12}}{Y_{12}^{0}}k_{12}\left(\frac{\dot{\varepsilon }}{{\dot{\varepsilon }}_{0}}\right)$$

To calibrate the constitutive model, only one parameter $Y_{33}^{0}/Y_{12}^{0}$ in the yield tensor Q need to be determined by a uniaxial compression and simple shear at the reference strain-rate (typically 0.001s^−1^). A series of uniaxial compression and simple shear tests at different strain rates were conducted to determine the elements $h_{ij}$ in hardening matrix ***H***. For simplification, the rate-dependent effects in compression and shear are assumed to be equal at low strain rates; i.e., $k_{11}=k_{12}$.

In practical applications, tension and compression may simultaneously occur in different regions of a component. Therefore, both responses are incorporated into a unified model and distinguished by the sign of the maximum principal strain $\varepsilon_{mp}$:

(30) $$\left\{\begin{array}{c} \varepsilon_{mp}\geq 0,\;Tension\\ \varepsilon_{mp}<0,\;Compression \end{array}\right.$$

Accordingly, a set of uniaxial tensile tests is also required to calibrate the yield tensor and hardening matrix for the tensile regime.

## 4. Prediction of Mechanical Performance of EPS Foam

To calibrate the parameters of the proposed constitutive model, uniaxial tension, compression, and shear tests were conducted. Subsequently, static three-point bending and dynamic impact experiments were performed to validate the predictive capability of the rate-sensitive constitutive model.

### 4.1. Experiments for Calibration of the Constitutive Model

#### 4.1.1. Experimental Procedures

**Figure 5:**
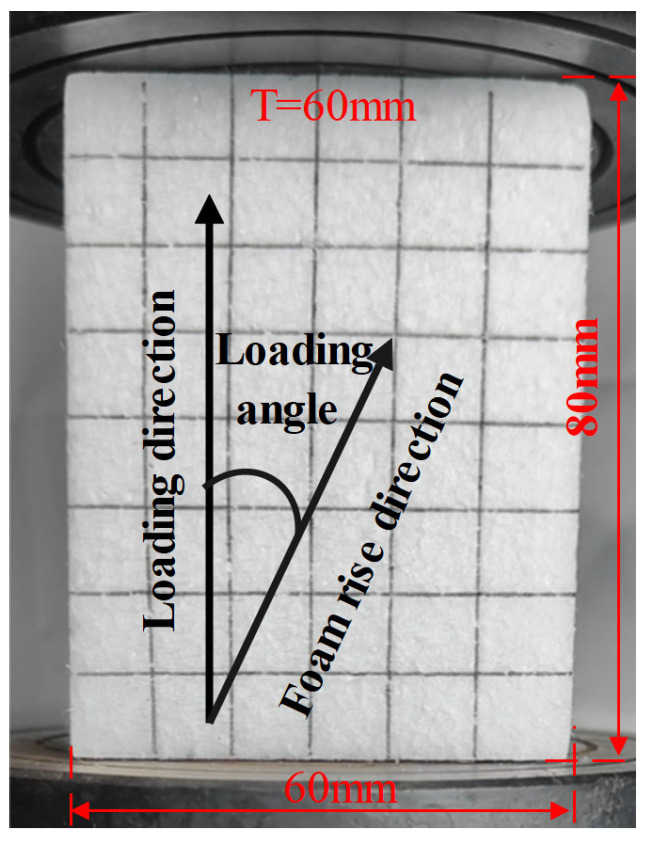
EPS foam specimen for calibration of the constitutive model.

Uniaxial compression tests were carried out using a universal testing machine (WDW-100M, Jinan Chenda Testing Machine Manufacturing Co., Ltd. in Jinan City, China). The loading velocities were varied to achieve different strain rates: 0.001 s^−1^, 0.01 s^−1^, and 0.05 s^−1^. To simulate high strain-rate loading conditions, dynamic compression tests at a strain rate of 55.9 s^−1^ were performed using a drop-weight impact tester (see Figure 1). The EPS foam specimens used in all tests had a density of 18.1 kg/m^3^ and were manually cut, which may introduce some unavoidable geometric inaccuracies. For each test condition, five specimens were prepared to ensure repeatability and minimize experimental errors. Figure 5 shows a typical compression specimen with dimensions of 60 mm × 60 mm × 80 mm. To examine possible anisotropy, compression loading was applied at different angles: 0°, 45°, and 90° relative to the specimen orientation.

**Figure 6:**
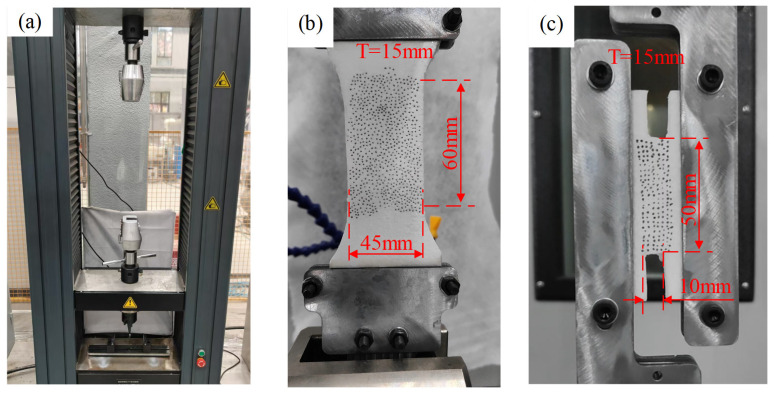
Experimental procedure: (**a**) universal testing machine fitted with tensile and compression load cells; (**b**) tensile test of dog-bone specimens with customed clamp; (**c**) shear test of butterfly-liked specimens with customed clamp.

In addition to compression tests, uniaxial tensile tests were conducted to characterize the engineering stress–strain response, particularly focusing on the brittle fracture behavior. Dog-bone-shaped specimens were clamped using a custom fixture, as shown in Figure 6b, and loaded at a constant rate corresponding to a strain rate of 0.001 s^−1^. The lower fixture remained stationary, while the upper fixture applied the tensile force.

Simple shear experiments were also performed using a universal testing machine. Butterfly-shaped specimens were clamped using a custom-designed fixture, as illustrated in Figure 6c. The left clamp was fixed, while the right clamp moved vertically at a constant velocity to induce shear deformation.

#### 4.1.2. Experimental Results

**Figure 7:**
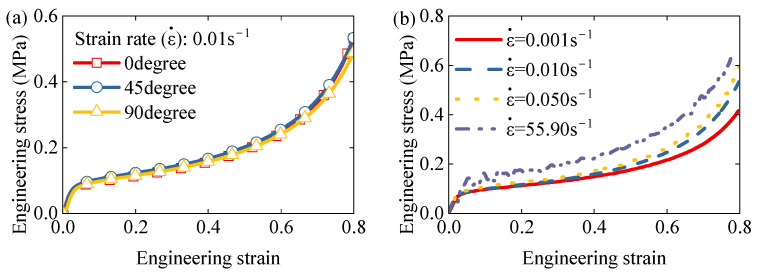
(**a**) Effect of loading angle on engineering strain–engineering stress at strain rate of 0.01s^−1^. (**b**) Effect of strain rate on engineering strain–engineering stress.

Figure 7a presents the engineering stress–strain curves for compression tests at various loading angles at a strain rate of 0.01 s^−1^. All curves exhibit comparable behavior during the elastic, plateau, and densification stages, suggesting negligible direction-dependence. Thus, the EPS foam can be reasonably assumed to behave as a quasi-isotropic material in subsequent modeling.

Figure 7b shows the effect of strain rate on the stress–strain response. Although the overall shape of the curves remains similar, increasing strain rates lead to higher yield stress and lower densification strain, indicating that EPS foam exhibits rate-dependent mechanical behavior.

**Figure 8:**
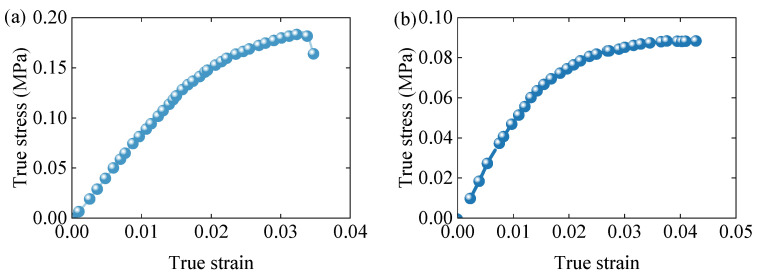
(**a**) True stress–true strain curve of uniaxial tensile response at strain rate 0.001 s^−1^. (**b**) True stress–true strain curve of shear response.

Figure 8a presents the tensile stress–strain curve at a strain rate of 0.001 s^−1^. The material demonstrates very limited plastic deformation before experiencing brittle fracture, with a fracture strain of approximately 0.035 and an ultimate tensile strength of about 0.18 MPa.

Figure 8b depicts the shear stress–strain response. Only the initial linear portion of the curve is considered valid due to potential inaccuracies in the nonlinear portion introduced by plastic deformation at the clamped edges and stress concentration at the specimen corners. These effects distort the nonlinear response, which is thus excluded from the model calibration.

#### 4.1.3. Calibration

Based on the experimental results from uniaxial compression, tension, and shear tests, the parameters of the quasi-isotropic constitutive model were calibrated and summarized in Table 1. To capture both compressive and tensile responses within a unified framework, the anisotropic yield tensor and hardening functions were calibrated separately. The dimensionless hardening functions $h_{ij}$ are provided in Table 2.

The parameters listed above were integrated into the proposed rate-sensitive constitutive model and implemented through the explicit user-defined subroutine VUMAT in ABAQUS. Since the subroutine operates in a local coordinate system while boundary conditions are defined in the global system, a second-order rotation tensor was used to transform between coordinate systems (for more details, see [1]).

### 4.2. Prediction of Static Three-Point Bending

**Figure 9:**
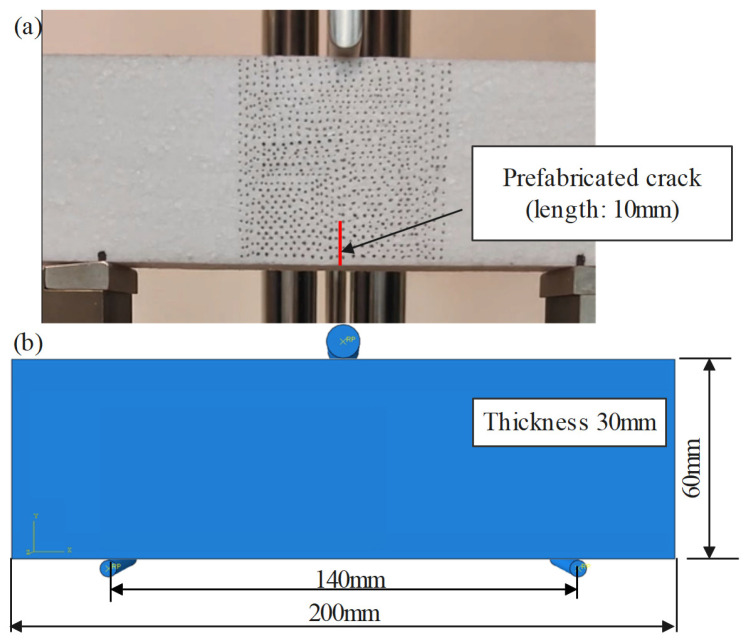
Experiment arrangement and simulation model for three-point bending test of EPS foam.

A cuboidal EPS foam specimen with dimensions of 200 mm × 60 mm × 30 mm was subjected to a three-point bending test using a universal testing machine equipped with a custom fixture, as shown in Figure 9. A prefabricated notch with a depth of 10 mm was introduced at the center of the specimen to study localized deformation. A constant loading velocity of 0.06 mm/s was applied to the upper loading head, producing a concentrated force at the midpoint. The foam rise direction coincided with the loading direction. The displacement and force of the upper loading head were collected by the in-built data acquisition in the machine.

To validate the model, a numerical simulation was carried out in ABAQUS using the same geometry and boundary conditions. The specimen was discretized using C3D8 brick elements with an element size of 2 mm, verified by a mesh sensitivity study. A constant loading velocity was applied to the upper surface, and mass scaling was used to improve computational efficiency. Contact interactions were defined using hard contact in the normal direction and frictional contact (coefficient = 0.3) in the tangential direction. The constitutive model was incorporated via the VUMAT subroutine. Note that since the explicit solver in ABAQUS does not directly support simulation of crack propagation, only the elastic and plastic response stages were simulated. The displacement and reaction force of the reference point in the top rigid body were collected to obtain the predicted force–displacement curve.

Figure 9 compares the three-point bending simulation with the experimental procedure, highlighting the presence of a prefabricated crack in the experimental specimens. Significant tensile strain localization will be observed at the mid-bottom region of the specimens. During the initial stages of the bending test, both elastic and plastic deformation occur, accompanied by crack propagation. In the experiments, this leads to a rapid reduction in structural stiffness once the crack initiates. While the simulation accurately captures the initial elastic and plastic responses, it does not account for crack-induced failure. As a result, plastic deformation continues in the simulation even after the experimental specimen fractures, leading to an overestimation of the stress.

**Figure 10:**
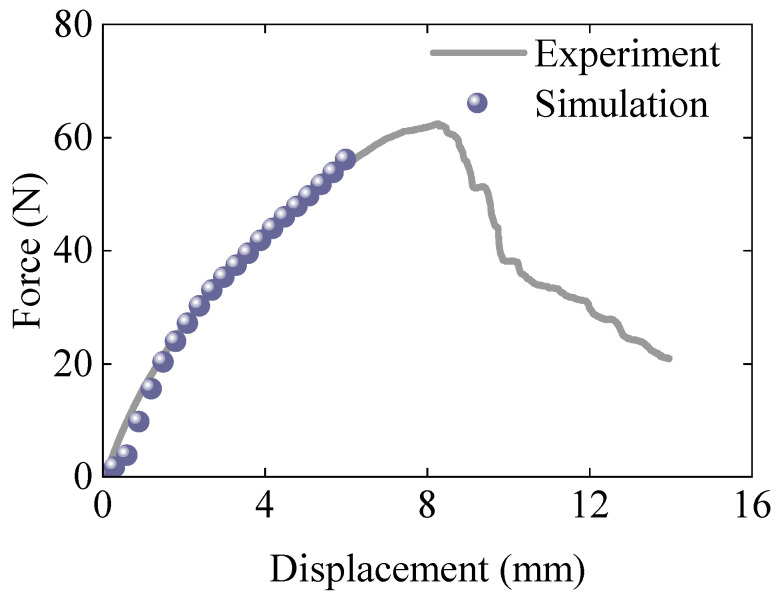
Comparison of force–displacement response between experimental and simulated three-point bending results.

The predicted result in Figure 10 exhibits a lower initial force compared to the experimental results. This discrepancy may be attributed to the smaller radius of the lower support in simulation, leading to higher stress concentration and reduced apparent stiffness compared to experimental results. Nevertheless, the proposed model accurately predicts the force–displacement response up to a displacement of 6 mm. Beyond this point, deformation is primarily driven by crack propagation, a mechanism not captured by the current model.

As predicted, the simulation model fails to capture the reduction in structural stiffness observed in the experiments due to crack propagation. Consequently, the simulated stress continues to increase during the plastic stage, diverging from the experimental results. Despite this limitation, the proposed constitutive model accurately reproduces the elastic and plastic behavior of the material, with the exception of crack propagation, which arises from the presence of prefabricated cracks in the experimental specimens.

### 4.3. Prediction of Dynamic Compression of Non-Standard Specimens

The standard and non-standard compressive specimens used in the experiments were also modeled in ABAQUS to simulate their dynamic compression behavior. The geometries were meshed using C3D8 solid elements with a global element size of 4 mm. A rigid plate was used to compress the specimens at an initial velocity of 4.5 m/s. Contact between the rigid plate and the foam was defined using a friction coefficient of 0.3 in the tangential direction and “hard” contact in the normal direction.

**Figure 11:**
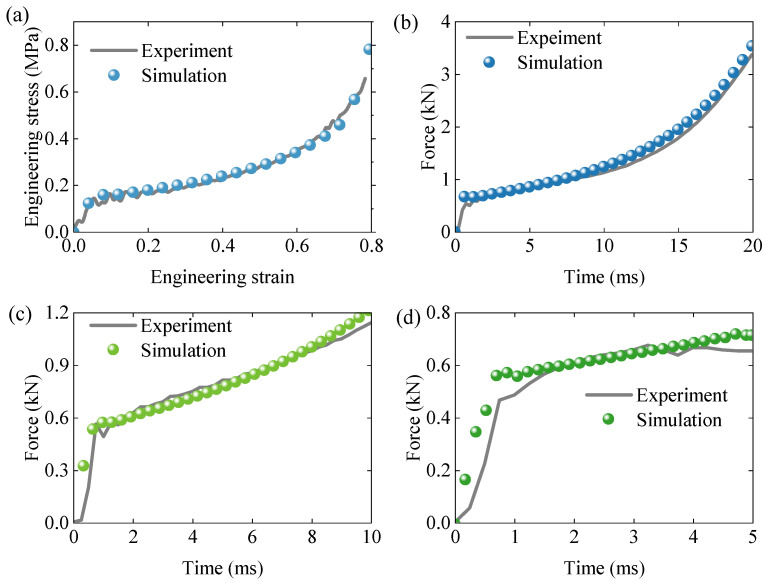
(**a**) Engineering stress–strain response of standard compression specimen. (**b**–**d**) Force–time curves of three typical non-standard compression specimens.

Figure 11a illustrates the engineering stress–strain response of the standard compression specimen. Good agreement is observed in both the initial elastic and subsequent plateau stages, indicating that the proposed constitutive model effectively captures the dynamic response of EPS foam. Figure 11b–d present the force–time curves for three additional non-standard specimens. While the model accurately predicts the early response phase, discrepancies appear at later stages due to differences in the deformation patterns between the simulations and experiments.

**Figure 12:**
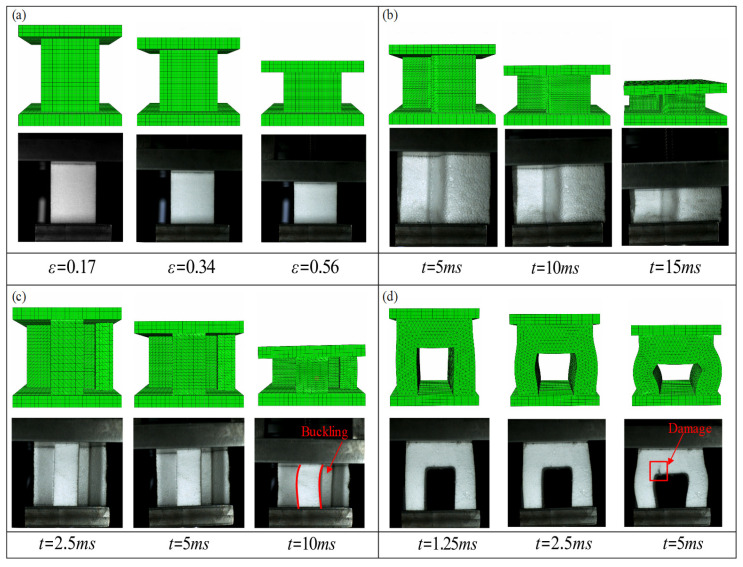
Deformation modes of EPS foam compressed under impact loading: (**a**) standard specimen; (**b**) non-standard specimen 1; (**c**) non-standard specimen 2; and (**d**) non-standard specimen 3.

In addition to force and strain responses, the deformation patterns observed in experiments and simulations are compared in Figure 12. Figure 12a,b show that for the standard and first non-standard specimen, the predicted deformation modes closely match experimental observations. In both cases, the deformation is uniform with negligible transverse expansion.

For non-standard specimen 2, shown in Figure 12c, in the initial stage (left), both simulation and experiment show that the specimen remains largely undeformed under low compressive load. The middle stage reveals uniform axial compression with minimal lateral displacement, indicating that the material is primarily undergoing elastic deformation and early-stage cell wall collapse. In the final stage (right) at *t* = 10 ms, localized lateral deformation is observed, particularly in the central web region of the component. This is evidenced by visible curvature and out-of-plane displacements in both the experimental and simulated results, consistent with a classical buckling mode due to the minimal height thickness ratio (85 mm/15 mm = 5.6). The region highlighted in red clearly marks the onset of buckling, demonstrating lateral instability due to the compressive stresses exceeding the critical load of the slender web section. In contrast to the experimental result, the simulated upper loading plate exhibits a slight tilt due to uneven contact force distribution, introducing noticeable differences between the two results.

When specimen 3 was subject to the compressive loading condition shown in Figure 12d, in the initial stage (left), both the numerical and experimental results show that the EPS component remains largely undeformed, indicating the onset of loading and uniform stress distribution. In the intermediate stage (middle), moderate deformation is observed primarily in the vertical struts of the component, where the foam begins to compress and local buckling effects start to emerge due to the cell wall bending and progressive collapse. The final stage (right) captures a pronounced deformation and localized failure. The experimental image clearly reveals damage initiation at the inner edge of the arch, identified by a red marker, resulting in lower contact forces that were not fully captured by the simulation. This damage is attributed to stress concentration and local densification, resulting in material fracture and crushing.

The proposed constitutive model accurately captured the deformation behavior of standard specimens. However, it exhibited computational discrepancies when applied to non-standard specimens, primarily due to unstable structural responses such as buckling in Figure 12c and fracture in Figure 12d. The primary objective of the model is to represent the elastic and plastic behavior of EPS foam structures. The favorable agreement with the experimental results demonstrates its strong potential for capturing complex deformation in large-scale structures, such as TV packaging, as discussed in the following section.

### 4.4. Prediction of Mechanical Performance of TV Package

A numerical model of the assembled TV package (which also includes a corrugated cardboard shell and a dummy TV model; see Section 2.2) was also established to predict the mechanical performance of EPS foam in TV packaging, as illustrated in Figure 3. The mechanical behavior of EPS foam was predicted using the proposed constitutive model via the explicit user subroutine VUMAT in ABAQUS, and the parameters used in this model were listed in Table 1 and Table 2. Other components, such as the corrugated board and the TV model, were given the proper material properties suggested by Hisense Virtual Technology Co., Ltd. (Qingdao, China), and these may be made available via direct contact with them. EPS foams were meshed using C3D4 tetrahedral elements with a global mesh size of 9 mm. Contact interactions among the components were defined using the “General Contact” algorithm in ABAQUS. Tangential behavior was modeled with a friction coefficient of 0.4, and normal behavior was set as “hard” contact. Loading conditions included an initial velocity of 3.1 m/s and a gravitational acceleration of 9.8 m/s^2^, applied to the entire model. Mass scaling was used to enhance computational efficiency.

**Figure 13:**
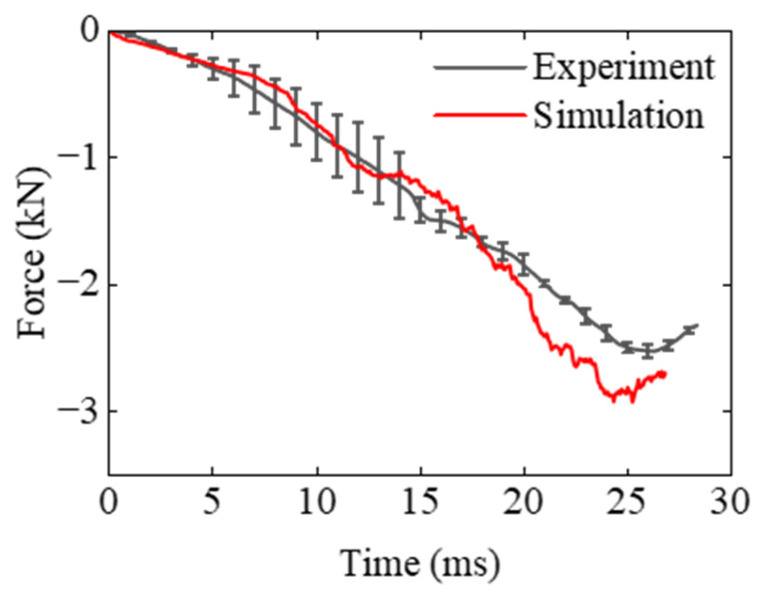
Force–time curve of large model impact response.

**Figure 14:**
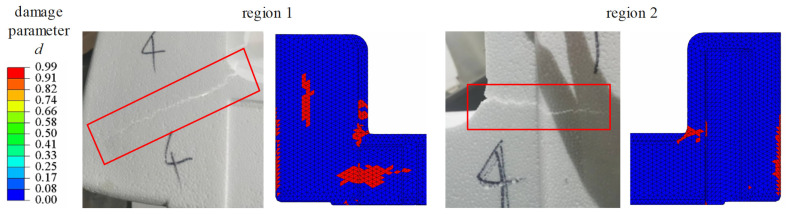
Comparison of damage distributions: simulation results at *t* = 30 ms vs. experimental post-impact observations.

The force shown in Figure 13 initially increases sharply upon impact, followed by a gradual decay as the EPS foam undergoes deformation and energy dissipation. The numerical results closely follow the experimental trend in the early phase of impact but diverge slightly in the later stage, possibly due to the complex contact behavior between components. The results also reveal a favorable correlation between simulation and experiment, indicating that the constitutive model captures the rate-sensitive mechanical response of EPS foam. The peak force predicted by the simulation is slightly higher than that observed experimentally. This discrepancy may result from localized damage near the corners, as illustrated in Figure 14.

Figure 14 shows that in region 1, both the experimental image and the simulation output reveal damage localization along a plane oriented at approximately 45°, indicative of shear-dominated failure. The close correspondence between the simulated damage elements and the experimentally observed crack path demonstrates the model’s capability to predict the orientation and distribution of shear cracks under dynamic loading. In region 2, damage propagation is predominantly horizontal, as evidenced by both the simulation and experimental results, further underscoring the model’s ability to capture the evolution of dominant failure paths in complex geometries. However, discrepancies between the simulated and experimental damage zones may arise due to several factors, including limitations in the material constitutive models (e.g., the TV model), inaccuracies in simulating interfacial contact, and inherent experimental variabilities.

## 5. Conclusions

The dynamic mechanical behavior of foam was systematically investigated through a combination of experimental testing and advanced numerical simulations, with a specific focus on both individual structural components and a fully assembled TV packaging system subjected to impact loading. A novel, rate-sensitive, isotropic constitutive model was developed and implemented, uniquely capable of capturing the foam’s complex response by integrating tensile and compressive behavior into a single unified framework. This model was rigorously validated using quantitative results from three-point bending and dynamic compression tests on specimens with diverse geometrical features, yielding excellent correlation with experimental measurements, and force–time curves and deformation from simulation matched experimental values. Notably, the simulations accurately predicted damage initiation and propagation paths in the assembled packaging structure under impact, faithfully reproducing observed crack angles, failure modes, and energy absorption levels. As a result, the presented constitutive model provides a robust and reliable tool for simulating the dynamic performance of EPS foam, and in particular, it provides a reference for the impact response of engineering structures consisting of EPS foam.

## Figures and Tables

**Table 1:** Values of elastic, yield and damage parameters for EPS foam.

Common				Compression			Tension				
ρ	$E_{S}$	$\upsilon^{e}$	$\upsilon^{p}$	$E_{C}$	$Y_{C}^{0}$	$Q_{1212}^{C}$	$E_{T}$	$Y_{T}^{0}$	$Q_{1212}^{T}$	$\varepsilon_{f}$	${\bar{u}}_{f}$
kg/m^3^	MPa	-	-	MPa	MPa	-	MPa	MPa	-	-	mm
18.1	4.6	0	0	3.5	0.085	1.82	6.4	0.112	1.07	0.04	1 × 10^−4^

**Table 2:** Dimensionless hardening functions for EPS foam.

**Compression**	$h_{11}=h_{22}=h_{33}$	$h_{12}=h_{23}=h_{13}$
	$\left(0.83+0.96{\bar{\varepsilon }}^{p}+0.17e^{1.67{\bar{\varepsilon }}^{p}}\right)\left(1+0.062\log_{10}\left(\frac{\dot{\varepsilon }}{{\dot{\varepsilon }}_{0}}\right)\right)$	$\left(1+3.19{\bar{\varepsilon }}^{p}\right)\left(1+0.062\log_{10}\left(\frac{\dot{\varepsilon }}{{\dot{\varepsilon }}_{0}}\right)\right)$
**Tension**	$h_{11}=h_{22}=h_{33}$	$h_{12}=h_{23}=h_{13}$
	$\left(1.84-0.858e^{-74.02{\bar{\varepsilon }}^{p}}\right)\left(1+0.062\log_{10}\left(\frac{\dot{\varepsilon }}{{\dot{\varepsilon }}_{0}}\right)\right)$	$\left(1+3.19{\bar{\varepsilon }}^{p}\right)\left(1+0.062\log_{10}\left(\frac{\dot{\varepsilon }}{{\dot{\varepsilon }}_{0}}\right)\right)$

## Data Availability

Data used in this work will be made available on proper request.
